# Entomological effects of attractive targeted sugar bait station deployment in Western Zambia: vector surveillance findings from a two-arm cluster randomized phase III trial

**DOI:** 10.1186/s12936-024-05045-3

**Published:** 2024-07-18

**Authors:** Joseph Wagman, Benjamin Chanda, Javan Chanda, Kochelani Saili, Erica Orange, Patricia Mambo, Rayford Muyabe, Tresford Kaniki, Mwansa Mwenya, Mirabelle Ng’andu, Jimmy Sakala, Willy Ngulube, John Miller, Annie Arnzen, Kafula Silumbe, Gift Mwaanga, Limonty Simubali, Alice Mungo, Monicah M. Mburu, Edgar Simulundu, Brenda Mambwe, Racheal Kasaro, Conceptor Mulube, Mulenga Mwenda, Busiku Hamainza, Ruth A. Ashton, Thomas P. Eisele, Angela F. Harris, Julian Entwistle, Joshua Yukich, Laurence Slutsker, Thomas R. Burkot, Megan Littrell

**Affiliations:** 1 PATH, Washington, DC USA; 2 PATH, Kaoma, Zambia; 3 PATH, Lusaka, Zambia; 4 PATH, Seattle, WA USA; 5 Macha Research Trust, Choma, Zambia; 6 National Malaria Elimination Centre, Lusaka, Zambia; 7 Centre for Applied Malaria Research and Evaluation, Tulane School of Public Health and Tropical Medicine, New Orleans, LA USA; 8 Innovative Vector Control Consortium, Liverpool, UK; 9 Independent Consultant, Atlanta, GA USA; 10 Australian Institute of Tropical Health and Medicine, James Cook University, Cairns, Australia

## Abstract

**Background:**

Attractive targeted sugar bait (ATSB) stations are a novel tool with potential to complement current approaches to malaria vector control. To assess the public health value of ATSB station deployment in areas of high coverage with standard vector control, a two-arm cluster-randomized controlled trial (cRCT) of Sarabi ATSB® stations (Westham Ltd., Hod-Hasharon, Israel) was conducted in Western Province, Zambia, a high-burden location were *Anopheles funestus* is the dominant vector. The trial included 70 clusters and was designed to measure the effect of ATSBs on case incidence and infection prevalence over two 7-month deployments. Reported here are results of the vector surveillance component of the study, conducted in a subset of 20 clusters and designed to provide entomological context to guide overall interpretation of trial findings.

**Methods:**

Each month, 200 paired indoor-outdoor human landing catch (HLC) and 200 paired light trap (LT) collections were conducted to monitor *An. funestus* parity, abundance, biting rates, sporozoite prevalence, and entomological inoculation rates (EIR).

**Results:**

During the study 20,337 female *An. funestus* were collected, 11,229 from control and 9,108 from intervention clusters. A subset of 3,131 HLC specimens were assessed for parity: The mean non-parous proportion was 23.0% (95% CI 18.2–28.7%, total n = 1477) in the control and 21.2% (95% CI 18.8–23.9%, total n = 1654) in the intervention arm, an OR = 1.05 (95% CI 0.82–1.34; p = 0.688). A non-significant reduction in LT abundance (RR = 0.65 [95% CI 0.30–1.40, p = 0.267]) was associated with ATSB deployment. HLC rates were highly variable, but model results indicate a similar non-significant trend with a RR = 0.68 (95%CI 0.22–2.00; p = 0.479). There were no effects on sporozoite prevalence or EIR.

**Conclusions:**

*Anopheles funestus* parity did not differ across study arms, but ATSB deployment was associated with a non-significant 35% reduction in vector LT density, results that are consistent with the epidemiological impact reported elsewhere. Additional research is needed to better understand how to maximize the potential impact of ATSB approaches in Zambia and other contexts.

*Trial registration number:* This trial was registered with Clinicaltrials.gov (NCT04800055, 16 March 2021).

**Supplementary Information:**

The online version contains supplementary material available at 10.1186/s12936-024-05045-3.

## Background

The massive scale-up of effective malaria vector control, largely insecticide-treated bed nets (ITNs) and to a lesser extent indoor residual spraying (IRS), across much of Africa since 2000 has been a tremendous public health success, averting more than 515 million cases of malaria between the years 2000–2015 [1, 2]. Subsequently, global progress towards further malaria reduction has stalled with persistent and residual transmission remaining significant problems in many communities [3–5]. New vector control tools and approaches that address key drivers of residual transmission, such as daytime and outdoor biting, outdoor resting, and insecticide resistance, are needed to complement current approaches [6].

Attractive targeted sugar bait (ATSB) is one promising new tool with potential to address several of these challenges by exploiting the natural sugar feeding behaviours of mosquitoes, complementing ITN and IRS approaches that exploit mosquito blood-feeding and resting behaviours [7]. ATSB is an attract and kill intervention intended to shorten malaria vector lifespans, reducing transmission by decreasing the probability that vectors live long enough to support parasite development and by suppressing overall vector population abundance, leading to reduced entomological inoculation rates (EIR) [8–10].

The Sarabi ATSB® station (Westham Ltd., Hod-Hasharon, Israel) has undergone multiple rounds of prototype refinement and field validation against malaria vectors in Israel and southern Mali, and has demonstrated significant reductions in *Anopheles sergentii* and *Anopheles gambiae *sensu lato (*s.l*.) population numbers, biting rates, and EIRs [11–13]. Although work in Mali continues to refine optimal ATSB station configurations and deployment strategies in the Sahel [14, 15], these initial results have also led to trials designed to evaluate ATSB impact on health outcomes in Mali and in other settings, including western Zambia and western Kenya [7].

Compared to southern Mali, western Zambia is more temperate, rainier, has more abundant natural sugar sources, greater mosquito diversity, and more dispersed housing patterns [16]. Nonetheless, a 2021 entomological validation study in Western Province, Zambia showed that natural populations of *Anopheles funestus s.l.* and *An. gambiae s.l.* each readily fed from Sarabi ASB stations (prototype ATSB stations with no toxicant in the bait) that were deployed on the external walls of sleeping structures [17]. These results supported the decision to implement a large-scale phase III cluster randomized controlled trial (cRCT) of Sarabi ATSB stations in Western Province Zambia [7], where malaria prevalence and incidence are high and the dominant vector species (*An. funestus *sensu stricto [*s*.*s*.]) opportunistically bites both indoors and outdoors, and during the night and early evening and morning hours [17].

The principal outcomes of the cRCT in Zambia were epidemiological measures to directly estimate the impacts of ATSB deployment on malaria case incidence and *Plasmodium falciparum* infection prevalence [18], and these results are reported in detail elsewhere (Ashton et al*.*, in review). This paper reports findings from the vector surveillance component of the trial, implemented in a subset of study clusters and designed to provide entomological context to help guide the overall interpretation of trial findings. The primary entomological outcome was the effect of ATSB station deployment on vector parity, with secondary outcomes being vector abundance, human biting rates, sporozoite positivity, and entomological inoculation rates (EIR) [7, 18].

## Methods

### Study site

A detailed description of the study area is provided elsewhere [16]. In brief, the study was conducted in communities across Nkeyema, Kaoma, and Luampa districts in Western Province, Zambia. Malaria transmission in Western Province is seasonal, with peak transmission typically from January to May, corresponding with the annual November to March rainy season. *Anopheles* spp. diversity is high, with at least 15 known species present [17]. *Anopheles funestus* s.s*.* is the dominant vector species in the study area, accounting for 95% of infectious bites during pre-trial entomological validation work; probable secondary vectors include *Anopheles arabiensis, Anopheles gambiae s.s.*, *Anopheles squamosus*, and *Anopheles coustani* [17]. Within the study area settlements are dispersed, with a median structure density of 0.36 (inter-quartile range = 0.19–1.24) per hectare in the cluster core sampling areas [16].

### Experimental design

A detailed description of the full trial protocol has been published previously [7]. In summary, a two-arm, cluster-randomized controlled trial (cRCT) measured epidemiological outcomes in 70 total clusters and entomological outcomes in a subset of 20 clusters. Before random allocation of clusters into one of the study arms, 30 clusters representative of each study district were selected for baseline mosquito collections from April to July 2021. On the basis of cluster accessibility, community acceptance of mosquito collection activities, and baseline mosquito abundance, 20 of these clusters were selected for inclusion as entomological surveillance clusters for the main trial. Restricted randomization was then used to assign all clusters in a 1:1 ratio to either the control (No ATSB) or intervention (ATSB) arm. Inclusion of mosquito collection activities as one of the restricted randomization criteria ensured that of the 20 entomological surveillance clusters, 10 were assigned to the control arm and 10 to the intervention arm. Additional restricted randomization criteria included baseline measures of ITN use, proportion of households reporting IRS in the previous year, number of households, malaria prevalence, and the presence of a health clinic within the cluster boundary. Housing construction characteristics are highly similar across Western Province [16] and were not considered during cluster randomization, though data on entomological surveillance household construction was collected and is available for future secondary analysis.

### Study outcomes

The primary entomological endpoint of the study was parity, i.e. the proportion of female *An. funestus s.l.* mosquitoes collected during human landing collection that were graded as non-parous during ovary dissection assessments (had never been gravid, sometimes referred to as the non-parous rate [NPR]), used as a proxy estimate of daily survival [18].

Key secondary outcomes included vector abundance assessed by CDC light trap collection, landing rate from human landing collection (as a proxy for human biting rate), sporozoite positivity (SP, the proportion of female vectors positive for circumsporozoite [CSP] antigens in the head and thorax during ELISA screening) estimated from a subsample of mosquitoes collected from both methods, and entomological inoculation rate (EIR, estimated from the HLC landing/biting rate and SP).

### Standard of care vector control

During the two-year trial period from November 2021 to June 2023, the Zambia National Malaria Elimination Programme (NMEP) adopted a mosaic approach to standard of care malaria vector control that aimed to achieve universal coverage of all households in Western Province with either ITNs or IRS. Each health facility catchment area (HFCA) received one of the interventions based on annual microplanning exercises that considered general campaign feasibility, housing density and structure suitability for spraying, and other operational factors [19]. Because study cluster boundaries were established without considering HFCA boundaries, most study clusters contained a mixture of households targeted for IRS and those targeted to receive ITNs.

IRS campaigns began in October of each year, utilizing a mixture of clothianidin and deltamethrin (Fludora® Fusion, Bayer). ITN distribution also occurred twice during the study period, from 25 February–17 March 2022 and again from 19 September to 14 October 2022. Due to high community demand, the first distribution provided one deltamethrin treated ITN (PermaNet® 2.0, Vestergaard) to each household across all study clusters regardless of IRS targeting. During the second distribution, only households not targeted to receive IRS were allocated one alphacypermethrin plus piperonyl butoxide ITN (Veeralin®LN, VKA Polymers) for every two household residents according to the Ministry of Health ITN distribution guidelines. As a result of the combined efforts, the trial site had high levels of coverage with standard of care vector control, with more than 70% of households having access to at least one ITN for every two residents or IRS [16].

### ATSB intervention

The Sarabi version 1.2 ATSB® station [20] evaluated here, which is described in detail elsewhere, incorporates a fruit syrup to attract sugar foraging mosquitoes mixed with the neonicotinoid insecticide dinotefuran (Mitsui Chemicals, Tokyo) as an ingestion toxicant to kill mosquitoes feeding from the bait station. During trial implementation, two ATSB stations were installed in intervention clusters on exterior walls of all eligible structures, defined as residential or cooking/kitchen structures where household members routinely slept, had at least three complete walls at least 1 m high, and a complete roof [7]. Installation campaigns were each conducted over two weeks just prior to the onset of the rainy season, from 1 to 13 November 2021 and again from 21 October to 12 November 2022. Additional descriptions and key details of ATSB deployment and monitoring activities implemented during the trial are provided elsewhere [20].

### Entomological surveillance

Monthly mosquito monitoring was conducted from November 2021 to June 2022 and again from November 2022 to June 2023, the periods when ATSB stations were deployed. Mosquitoes were collected by human landing catch (HLC) and CDC Miniature Downdraft Blacklight UV Light Traps (CDC LT) (Model 912, John W. Hock Co., Gainesville FL). Each month, 20 households were selected for mosquito collection in each entomological surveillance cluster. The sampling frame for the entomological collections was a modified list of households enumerated during the pre-trial census. To assist with household identification and consenting, households from the master list that were eligible for entomological surveillance were limited to those for which the name of the head of household was recorded during the census, and to minimize inconvenience households were not eligible to be sampled in two consecutive months. From this list, which was updated monthly, ten households were randomly selected for paired indoor-outdoor HLC collections, and 10 nearby households (neighbours) were identified for paired indoor-outdoor CDC LT collections. Households were sampled for one night each, resulting in 200 paired indoor-outdoor CDC LT and 200 paired indoor-outdoor HLC collection nights per month. Prior to each month’s collection schedule, households selected for vector surveillance were visited and consent from the head of household obtained. To help prevent contamination, a ‘fried egg’ study design was used and collections were made only from households within the cluster core, which was surrounded by a 600 m buffer zone in which all households received the corresponding ATSB and standard of care vector control interventions (intervention clusters) or just standard of care vector control interventions (control clusters) but were not included in entomological data collection activities.

During paired HLC collections, indoor collectors were seated inside a household sleeping structure, in a room adjacent to the building entrance with the door closed. Outdoor collectors were seated outside the same structure, in the peridomestic space a minimum of 5 m and a maximum of 10 m from the structure door. Mosquito collections were made for 12 h, from 18:00 h until 06:00 h the next morning. For 45 min of every hour, collectors used a flashlight to scan their exposed legs for host-seeking mosquitoes. Mosquitoes seen on or near their legs, or felt alighting on their skin, were collected via mouth aspiration and placed into the corresponding pre-labelled collection cup. The last 15 min of each collection hour were used for resting, data entry, and preparing for the next collection.

Indoor CDC LTs were hung near the foot of an occupied sleeping space, with the trap fan approximately 1.5 m above the floor. Outdoor CDC LTs were also situated with the trap fan approximately 1.5 m above the ground, hung 5-10 m from the entrance to the structure in a location at least partially protected from the weather (for example, utilizing household eaves) but not in the direct vicinity of any ATSB stations (i.e. not positioned along the same wall). Appropriately labelled CDC LT collection cups were installed and light traps were switched on at 18:00 h and operated continuously for 12 h until 06:00 h the next morning.

As part of a national COVID-19 mitigation strategy, indoor HLC collections were suspended temporarily from late December 2021 through most of January 2022–part of collection months 3 and 4. Although no indoor HLC collections for months 3 and 4 are included in the analysis, outdoor HLC and all CDC LT collection results are included.

Insecticide resistance profiles to alphacypermethrin, deltamethrin, permethrin, and pirimiphos-methyl were determined using standard WHO tube bioassay tests against local adult female *An. funestus s.l.* collected via Prokopack aspiration [21]. A standardized topical application bioassay was used to evaluate susceptibility to orally ingested dinotefuran (the active ingredient in the ATSB station) [22].

### Sample processing

At the conclusion of each night’s collection, all labelled collection cups were placed in appropriately labelled cooler boxes humidified with a damp towel for transport to the field laboratory in Kaoma town.

Mosquitoes collected during HLC and arriving to the laboratory alive were rapidly knocked down via mechanical shaking of the collection cup, and all anopheline mosquitoes were morphologically identified to species or species group [23]. For each anopheline mosquito collected, household, time, date, indoor/outdoor location, and method of collection was recorded. Each anopheline was also classified based on abdominal appearance as unfed, partly fed, fed, or gravid [24]. Freshly killed, not gravid and not blood-fed *An. funestus s.l* and *An. gambiae s.l.* mosquitoes were assessed for parity using standard ovary dissection techniques [24, 25], with the remaining mosquito carcass placed on silica gel in individual 1.7 ml Eppendorf microcentrifuge tubes (Sigma co. Ltd) labelled with a sample ID. Parity assessments were scored independently by two technicians with any discrepant results resolved by an entomology supervisor.

In the lab, anopheline specimens from CDC LT collections were killed in a − 20 °C freezer, sorted by genus, identified to species or species group and characterized by abdominal appearance, as above [23, 24]. Household, time, date, location (indoor/outdoor), and method of collection of each anopheline collected was recorded. Specimen ID and storage was as performed as described above.

A representative sub-sample of just over 14,300 *Anopheles* spp. mosquitoes collected were further selected for PCR species identification and ELISA sporozoite screening. Samples were randomly selected from among pools designed to ensure a minimum of 4,000 *An. funestus s.l.*, and 1,000 each of *An. gambiae s.l.*, *Anopheles coustani s.l., Anopheles squamosus*, and other *Anopheles* spp., balanced across study months, study clusters, trap locations, and collection methods. For species identification, DNA was extracted from the specimen’s abdomen, legs, and/or wings and individually amplified using the appropriate *An. gambiae* [26] and/or *An. funestus* [27] PCR assays. The head and thorax of each specimen was then tested for the *P. falciparum* circumsporozoite protein (CSP) [28].

### Data collection tools and data storage

Data describing each participating household, mosquito collection point, and anopheline mosquito specimen collected were recorded on Android tablet devices using study specific CommCare (Dimagi Inc., Cambridge MA) data collection tools. Individual anopheline mosquitos were linked to their corresponding household, date, time, collection technique and indoor/outdoor location of capture by unique barcode IDs. Automated CommCare-created Excel dashboards were used for weekly data review and quality checks. CommCare datasets were exported to Microsoft Excel (Microsoft Corporation, Redmond, WA) and cleaned, transformed, and summarized by descriptive statistics (including species compositions and abundance) using Excel and Tableau Desktop v2020.4.11 (Tableau Software LLC, Seattle, WA).

### Statistical and analytical approaches

Full details of the trial statistical analysis plan are available elsewhere [18]. Briefly, based in part on mosquito densities collected during the pre-trial feeding validation study, it was estimated that sampling monthly over 7 months, from 10 entomological surveillance clusters per study arm (20 total clusters), at 10 households per month per cluster (200 total households per month) would yield 80% power (β = 0.80) to detect an increase in non-parous proportion from 48.8% to 57.8% with a two-tailed α = 0.05. This is roughly equivalent to a reduction in the probability of daily survival from 80% in the control arm to 75% in the intervention arm [29]. Key assumptions included an inter-month variance of 1 (giving a monthly mean non-parous proportion range of approximately 0.2 to 0.8), and a mean total number of 2.5 female mosquitoes collected via HLC per household per night [18]. Most assumptions used in the calculation of these estimates, with the exception of the mean number of female mosquitoes collected during HLC, were based upon the previous ATSB validation and modelling work and the anticipated impact of background interventions on mosquito survival. Stata/SE 14.2 (StatCorp LLC, College Station, TX) was used for the statistical analyses. Proportion (parity and sporozoite positivity) and mean (vector abundance and landing rates) confidence intervals were calculated using robust standard errors adjusted for clustering at the study cluster level [*vce(cluster)* command in Stata/SE].

Vector parity was assessed using specimens collected during HLC to ensure that fresh samples in good physical condition were available for ovarian dissections. The proportions non-parous were compared across study arm using a mosquito-level, multi-level generalized linear model (GLM) with a Bernoulli likelihood and a logit link function, including random intercepts for each cluster and household. Secondary adjusted analyses considered fixed effects for collection location (indoor/outdoor), time since ATSB deployment, and calendar month. Results are presented as an odds ratio.

Vector abundance, using household-level aggregate data from indoor and outdoor CDC LTs, was compared across study arms using a GLM with a Poisson likelihood and a log link function with random intercepts included for each cluster, and results presented as a rate ratio. Human landing rates from HLC were similarly compared using a GLM with a Poisson likelihood and a log link function with random intercepts included for each cluster, and results presented as a rate ratio.

Sporozoite positivity was analysed using a mosquito-level, multi-level GLM with a Bernoulli likelihood and a logit link function, with random intercepts for each study cluster. Secondary adjusted analyses considered fixed effects for collection method, collection location (indoor/outdoor), time since ATSB deployment, and calendar month. Results are presented as an odds ratio. To maximize the number of mosquitoes available for CSP ELISA screening, specimens from both collection methods were tested.

Comparisons of calculated EIR across study arms were based on Student’s *t*-test, and used estimates of EIR made independently for each study cluster using the results of the HLC and sporozoite positivity analyses to calculate a measure of the number of infectious bites per person per month.

### Ethical considerations

Written informed consent was collected from the heads of all households where mosquito surveillance activities took place. Mosquito collectors and supervisors were trained community members. As per guidance from community leadership, indoor HLC collections were only conducted in structures where sleeping residents were the same gender as the mosquito collector. All HLC collectors were tested monthly for malaria (Bioline™ Malaria Ag Pf Rapid Diagnostic Test [RDT], Abbott Diagnostics), prior to each collection round. Individuals who were RDT positive received a standard course of of 80 mg artemether/480 mg lumefantrine (LonArt®, Bliss GV Pharma Ltd) and did not participate in that month’s collection. Collectors testing RDT negative received two standard tablets of 12.5 mg dapsone/100 mg pyrimethamine (Deltaprim™, Zimbabwe Pharmaceuticals Ltd) as malaria chemoprevention. This study received ethical approval from the University of Zambia Biomedical Research Ethics Committee (Ref # 1197–2020), the PATH Research Ethics Committee (Ref # 1,460,046–5) and Tulane University (Ref # 2019–595), and is registered at ClinicalTrials.gov (NCT04800055).

## Results

A total of 120,580 *Anopheles* spp. mosquitoes were collected, with slightly fewer collected in study year two (53,536; 44% of total) compared to year one (67,044; 56%). More *Anopheles* spp. were collected using HLC (74,201; 60%) than CDC LTs (48,379; 40%). Of those *Anopheles* spp. for which the sex of the specimen was determined (120,249; 99.7%), more than 97% (117,107) were female and 2.6% (3142) were male, and more than 92% of the male specimens (2899) were collected in CDC LTs. The most abundant species morphologically identified was *An. squamosus* (37,203; 31%), followed by *An. funestus s.l.* (22,987; 19%), *Anopheles tchekedii* (22,755; 19%) *An. coustani s.l.* (19,669; 16%), *Anopheles maculipalpis* (5,786; 5%), *Anopheles tenebrous* (4730; 4%), *An. gambiae s.l.* (2721; 2%), and *Anopheles gibbinsi* (2312; 2%).

A total of 14,306 specimens from ten different species or species groups were screened for *P. falciparum* CSP antigens in the head and thorax (Table 1). Hereafter, mosquitoes positive for the *P. falciparum* CSP antigen are referred to as either being sporozoite positive or as being infectious. The specimens sub-sampled for ELISA analysis were also identified to species by PCR. There were 165 positive specimens, 96.4% of which (159) were *An. funestus s.l.* Other *P. falciparum* positive species included *An. coustani s.l.* (3), *An. gambiae s.l.* (2), and *An. squamosus* (1).

**Table 1:** Results from the *P. falciparum* CS ELISAs

Species	*P. falciparum* ELISA results						
	Negative	Positive	Crude SP	% of all sporozoite positives (N = 165)	Overall number of female mosquitoes collected^a^	Estimated number of infectious mosquitoes collected	Estimated % of all infectious mosquitoes (N = 716)
*An. funestus s.l*	4609	159	3.3%	96.4	20,337	678	94.7%
*An. coustani s.l*	2500	3	0.1%	1.8	19,564	23	3.3%
*An. gambiae s.l*	1122	2	0.2%	1.2	2642	5	0.7%
*An. squamosus*	3868	1	0.03%	0.6	36,881	10	1.3%
*An. gibbinsi*	3	0	–	–	2287	–	–
*An. maculipalpis*	1979	0	–	–	5765	–	–
*An. pretoriensis*	3	0	–	–	12	–	–
*An. rufipes*	41	0	–	–	163	–	–
*An. tchekedii*	13	0	–	–	22,665	–	–
*An. tenebrosus*	3	0	–	–	4645	–	–
Total	14,141	165	–	100%	114,961	716	100%

*SP* Sporozoite Positivity (proportion positive out of all tested); ^a^Total number of females collected in CDC LTs and HLC

While most infectious bites would have occurred indoors (61.5%), outdoor transmission also likely occurred during the study (Table 2).

**Table 2:** Location of infectious mosquito capture by species (combined CDC LT and HLC results)

Species	Estimated number of infectious mosquitoes^1^ (% of all infectious mosquitoes)	
	Indoors N (%)	Outdoors N (%)
*An. funestus s.l*	421 (58.8)	257 (35.9)
*An. coustani s.l*	14 (2.0)	9 (1.3)
*An. gambiae s.l*	5 (0.7)	0 (0.0)
*An. squamosus*	0 (0.0)	10 (1.4)
Total	440 (61.5)	276 (38.6)

^1^Location specific proportion sporozoite positive multiplied by the total number of mosquitoes collected in that location

Within the *An. funestus* group, 93.5% (3287) of specimens were confirmed by PCR as *An. funestus s.s*.–including all CS-positive specimens. Other species identified include *Anopheles leesoni* (126), *Anopheles parensis* (90), *Anopheles rivulorum*-like (6), *Anopheles longipalpis* (4), *An. funestus*-like (4), *Anopheles rivulorum* (3), and *Anopheles vaneedeni* (2).

Within the *An. gambiae* complex, 97.7% (1042) of morphologically identified specimens were confirmed to species by PCR as *An. arabiensis*, 1.7% (18) were *An. gambiae* s.s., and 0.7% (7) were *An. quadriannulatus*. Of the two sporozoite positive specimens from this complex, one was *An. arabiensis* and one was *An. gambiae s.s*.–both of which were collected indoors. Subsequent entomological results and analysis presented here focus on female *An. funestus s.l.*, the clearly dominant vector species in the study area during the trial, though it should be noted that *An. coustani s.l.*, *An. gambiae s.s*., *An. arabiensis*, and *An. squamosus* may also have played a minor role in malaria transmission as secondary vectors.

Standard WHO tube bioassay tests [21] using locally captured *An. funestus s.l.* adult females from the study site indicated high levels of resistance to the pyrethroids alphacypermethrin, deltamethrin, and permethrin, with 24-h mortality ranging from 46 to 60% [16]. Resistance was not detected to pirimiphos-methyl, nor to dinotefuran using the topical assay [22]**.**

During both study implementation years high ATSB station coverage was reported across all intervention clusters: among eligible structures assessed, 93.1% (95%CI 91.6–94.7%) had two ATSB stations in any condition hanging and 71.5% (95%CI 67.1–75.8%) had two ATSB stations in good condition hanging [20]. While programmatic data and outcome indicators from the IRS and ITN campaigns cannot be aligned with the study clusters, data from the annual cross-sectional surveys (Ashton et al*.*, in review) confirm that the entomological surveillance clusters were balanced in terms of the proportion of households having received IRS within the previous year, the proportion of households that owned one net per two persons, and the proportion of household residents that slept under a net the previous night (Table S1).

A total of 3,131 female *An. funestus* specimens collected by HLC were successfully assessed for parity status, 1,477 from control clusters and 1,654 from intervention clusters (Table 3). The mean non-parous proportion was 23.0% (95% CI 18.2%–28.7%) in the control arm and 21.2% (95% CI 18.8–23.9%) in the intervention arm, resulting in an unadjusted OR = 1.05 (95% CI 0.82–1.34; p = 0.688). The non-parous proportion and the total number of mosquitoes dissected were both higher in year 2, but there were no significant differences between study arms in either year. Covariate adjusted analysis accounting for the number of months since ATSB deployment, calendar month (as a seasonal adjustment), and location of mosquito capture (indoors or outdoors) did not change the effect estimate of ATSB deployment on *An. funestus* non-parous proportion (OR = 1.00 [95% CI 0.78–1.24, p = 0.914]).

**Table 3:** Proportions non-parous for *An. funestus* collected during HLC activities

	Control arm		Intervention Arm		Primary unadjusted OR	
	Number of *An. funestus* assessed	Proportion non-parous	Number of *An. funestus* assessed	Proportion non-parous	OR	*p*
		(95% CI)*		(95% CI)*	(95% CI)	
Year 1	181	13.3%	118	14.4%	0.90	0.826
		(6.2–26.1%)		(11.0–18.6%)	(0.35–2.33)	
Year 2	1296	24.4%	1536	21.7%	1.10	0.453
		(19.9–29.5%)		(19.4–24.3%)	(0.86–1.42)	
Overall	1477	23.0%	1654	21.2%	1.05	0.688
		(18.2–28.7%)		(18.8–23.9%)	(0.82–1.34)	

n = number of female *An. funestus* assessed as non-parous; N = total number of female *An. funestus* assesses for parity

*Robust standard errors accounting for clustering effects

A total of 12,206 female *An. funestus* were collected in CDC LTs during the study: 7,305 from control clusters and 4,901 from intervention clusters (Table 4). The mean number of *An. funestus* mosquitoes collected per household per night (indoor and outdoor traps) was 4.8 (95% CI 2.5– 7.0) in the control arm and 3.2 (95% CI 1.5–4.9) in the intervention arm, resulting in an unadjusted RR of 0.65 (95% CI 0.30–1.40, p = 0.267). In general, *An. funestus* abundance was higher in year two than in year one and indoors compared to outdoors, but there were no significant differences in *An. funestus* abundance across study arms (Table 4). Covariate adjusted analysis accounting for the number of months since ATSB deployment and calendar month did not result in any change in ATSB effect size or significance (RR of 0.64 (95%CI 0.30–1.40, p = 0.265). Study arm specific light trap densities by cluster, by year, and by month are presented in Figure S1.

**Table 4:** Mean number of *Anopheles funestus* females collected in CDC LTs

	Control arm		Intervention Arm		Primary unadjusted RR	
	N (Trap nights)	Mean *An. funestus* per trap night	N (Trap nights)	Mean *An. funestus* per trap night	RR	*p*
		(95% CI)*		(95% CI)*	(95% CI)	
Year 1						
Indoor	782	2.6	778	1.6	0.71	0.453
		(0.9–4.3)		(0.4–2.8)	(0.28–1.74)	
Outdoor	782	1.3	778	0.6	0.50	0.198
		(0.6–2.1)		(0.3–1.1)	(0.18–1.43)	
Total	1564	3.9	1556	2.3	0.65	0.361
		(1.5–6.4)		(0.9–3.8)	(0.25–1.64)	
Year 2						
Indoor	752	4.2	745	3.1	0.64	0.213
		(2.6–6.0)		(1.5–4.7)	(0.31–1.29)	
Outdoor	752	1.3	745	1.0	0.70	0.306
		(0.9–1.8)		(0.6–1.4)	(0.36–1.38)	
Total	1504	5.6	1490	4.1	0.65	0.232
		(3.5–7.8)		(2.1–6.1)	(0.32–1.31)	
Overall						
Indoor	1534	3.4	1523	2.3	0.66	0.277
		(1.7–5.1)		(1.0–3.7)	(0.31–1.40)	
Outdoor	1534	1.3	1523	0.8	0.61	0.242
		(0.7–1.9)		(0.5–1.2)	(0.27–1.39)	
Total	3068	4.8	3046	3.2	0.65	0.267
		(2.5–7.0)		(1.5–4.9)	(0.30–1.40)	

*Robust standard errors accounting for clustering effects

A total of 8,131 female *An. funestus* were collected during HLC: 3,924 from control clusters and 4,207 from intervention clusters (Table 5). As was noted with CDC LT abundance (Table 4), mosquito human landing rates were higher in year two than in year one, though rates were higher outdoors in year one and indoors in year 2. The mean number of mosquito landings per household per night (indoor and outdoor) was 2.5 (95% CI 1.2–3.8) in the control arm and 2.7 (95% CI 0.59–4.8) in the intervention arm. Despite the highly similar mean landing rates across study arms, the modeled unadjusted RR is 0.68 (95%  CI 0.23–2.00, p = 0.479), which, while not statistically significant, does suggest an overall trend towards fewer mosquitoes per night in the ATSB arm when accounting for the effects of clustering. These discordant results are likely because of the substantial variation in HLC landing rates observed between clusters (intercluster coefficient of variation = 0.948), months, and years (Fig. S2). Covariate adjusted analysis accounting for the number of months since ATSB deployment, calendar month, and HLC collection team did not result in any change in ATSB effect size or significance (RR of 0.65 [95% CI 0.22–1.89, p = 0.426]).

**Table 5:** Female *Anopheles funestus* landing rates during HLC

	Control arm		Intervention Arm		Primary unadjusted RR	
	N (Trap nights)	Mean *An. funestus* per collection night	N (Trap nights)	Mean *An*.* funestus* per collection night	RR	*p*
		(95% CI)*		(95% CI)*	(95% CI)	
Year 1						
Indoor	784	0.68	782	0.34	0.60	0.534
		(0.21–1.2)		(0.10–0.60)	(0.11–3.04)	
Outdoor	784	1.1	782	0.69	0.49	0.286
		(0.39–1.9)		(0.13–1.2)	(0.13–1.83)	
Total	1568	1.8	1564	1.0	0.53	0.370
		(0.61– 3.0)		(0.27–1.8)	(0.13– 2.13)	
Year 2						
Indoor	782	2.1	784	2.5	0.58	0.350
		(0.96– 3.3)		(0.29–4.7)	(0.18–1.83)	
Outdoor	782	1.1	784	1.8	0.84	0.792
		(0.39–1.7)		(0.38–3.2)	(0.23–3.06)	
Total	1564	3.2	1568	4.3	0.64	0.475
		(1.4–5.0)		(0.68–8.0)	(0.19–2.16)	
Overall						
Indoor	1566	1.4	1566	1.4	0.56	0.320
		(0.71–2.1)		(0.22–2.6)	(0.18–1.75)	
Outdoor	1566	1.1	1566	1.3	0.82	0.730
		(0.46–1.7)		(0.35–2.2)	(0.27–2.50)	
Total	3132	2.5	3132	2.7	0.68	0.479
		(1.2–3.8)		(0.59–4.8)	(0.23–2.00)	

*Robust standard errors accounting for clustering effects

A total of 159 of 4,767 *An. funestus* specimens were positive for *P. falciparum* sporozoites (Table 1) (an overall crude SP = 3.3%). The mean sporozoite positivity was 2.7% (95% CI 1.7–4.1%) in the control arm and 4.1% (95% CI 3.0–5.5%) in the intervention arm, with an unadjusted OR of 1.55 (95% CI 0.93–2.57, p = 0.090). Covariate adjusted analysis accounting for the collection method and location (indoor or outdoor), as well as the number of months since ATSB deployment and the calendar month, did not result in any change in ATSB effect size or significance (OR = 1.57 [95% CI 0.94–2.63, p = 0.088]).

To compare EIRs across the study arms, cluster-specific estimates of SP and human landing rate for *An. funestus s.l.* were used to independently calculate estimated EIRs for each cluster (Table S2). The overall monthly EIR for *An. funestus s.l.* was 4.6 (95% CI 2.4–6.7) infectious bites per household per month in the control arm and 7.4 (95% CI 3.0–11.7) in the intervention arm, a non-significant difference of 2.8 infectious bites per household per month (95% CI − 7.3–1.7, p = 0.216).

## Discussion

Reported here are entomological findings from the first large-scale randomized trial of ATSB for malaria control. Vector surveillance was conducted in a subset of 20 of the total 70 trial clusters, and was designed to provide entomological context to help guide the overall interpretation of main epidemiological trial findings. Although the ATSB intervention was well implemented and achieved high coverage of eligible structures with two bait stations, the expected effect of ATSB deployment on *An. funestus* parity was not observed. The study was designed to detect an increase in the proportion of *An. funestus* that were non-parous from 48.8 to 57.8%, but the non-parous proportions observed across the study area showed little variability and were substantially lower than expected at 21.2% in the intervention arm and 23.0% in the control arm, a non-significant difference (p = 0.688).

Although not statistically significant (p = 0.267), trends in vector abundance as measured in CDC LT collections were more encouraging, indicative of a 35% relative reduction in *An. funestus* abundance associated with ATSB deployment. A similar trend was suggested by analysis of the HLC data, with the landing rate ratio indicating a non-significant (p = 0.479) 32% reduction in *An. funestus* bites. This trend was somewhat counterintuitive given that the crude estimates of average bites per household per night were nearly equivalent in the intervention and control clusters (Table 5; Fig. S2a), though the wide confidence intervals around these point estimates indicate high uncertainty around their precision. In fact, during year one the mean rates were in line with the overall modelled rate ratio (Fig. S2b), but in year two there were multiple outlier nights with greater than 62 *An. funestus s.l.* bites recorded at a single household, which accounts for the overall means being out of line with the rate ratio estimate (Fig. S2).

The moderate, albeit non-statistically significant, decrease in vector abundance aligns with the primary epidemiological outcomes of the trial (Ashton et al*.*, in review), which indicated modest, non-significant decreases in malaria case incidence (9% reduction) and prevalence (5.2% reduction) associated with ATSB deployment.

*Anopheles funestus* sporozoite positivity and EIR were both highly variable across clusters, with no significant differences observed across intervention and control arms. Considering the challenges involved in accurately and precisely measuring sporozoite rates [30–34], these results are not unexpected. It should also be noted that while EIR estimated from the HLC methods used during this study provide reasonable estimates of typical malaria exposure in and around households during nighttime hours, this may not be indicative of the entirety of community exposure. Similarly, no significant differences were observed for the primary parity outcome, where the higher-than-expected proportions parous observed across all study clusters suggests relatively long-lived *An. funestus* populations in the study site. Similar observations were reported recently in Tanzania, where on average *An. funestus* live twice as long and fly further than sympatric *An. arabiensis* [35]. Despite a robust entomological sampling effort embedded within a well-designed and implemented randomized trial, it is likely that the study was underpowered to detect small differences in these indicators–a significant challenge for many (if not most) entomological assessments of vector control impact [36–39].

Results from the pre-trial entomological validation study suggested that *An. funestus* vector populations in western Zambia readily fed from Sarabi bait stations at an estimated 8.6% daily feeding rate [17], which was expected to achieve significant reductions in both vector survival and malaria incidence [8, 9]. The entomological results presented here, as with the epidemiological results reported by Ashton et al*.* (in review), did not meet these expectations. It is interesting to note that the lower than assumed proportions of *An. funestus* that were non-parous observed during the study indicate that these malaria vector populations in Zambia are longer lived, with higher daily survival rates, than the *An. gambiae s.l.* populations from Mali used to calibrate some of the statistical assumptions when designing this trial [7, 18] and which informed the modelling of expected results of various ATSB interventions [8, 9, 15]. Furthermore, while *An. funestus* is generally known to breed in semi-permanent water bodies with algae and other aquatic vegetation, its specific larval habitats and breeding locations are difficult to identify [40, 41] and are poorly characterized in Western Zambia [42]. As such, *An. funestus* larval habitats are likely to be some distance away from the households where blood meals are typically acquired, and where ATSBs were hung during this trial. How these mosquito movements, and the availability of natural sugar sources in various locations, might influence vector sugar feeding behaviours is unknown and should be considered during future studies. It is possible that the specific bait station feeding rates required to achieve disease reduction outcomes might be different in different transmission settings, and higher bait station feeding rates may be required in settings with abundant sugar sources, highly efficient long-lived vectors, high malaria burdens, and dispersed housing patterns.

The spatial density of ATSB stations achieved during deployment may be an important determinant of potential impact on entomological and epidemiological outcomes. An analysis of epidemiological trial outcomes within subgroups defined by relatively low structure density (less than one structure per hectare) versus relatively high structure density (one or more structures per hectare) suggested that bait station density may be associated with outcomes of interest (Ashton et al*.*, in review). Subgroup analyses were not feasible within the entomological surveillance study due to the relatively small number of entomological surveillance clusters (20) compared with the total number of trial clusters (70). Of note, the pre-trial feeding study was conducted in study clusters with a higher structure density (median 0.72 structures per hectare) compared with the main trial (median 0.36 structures per hectare), and the ATSB research that preceded the Zambia trial and informed models to predict potential impact were based on settings in Mali with substantially higher structure densities. Attract-and-kill pest control interventions typically define target spatial densities or application rates (e.g., lures or grams of active ingredient per hectare) required to achieve desired impact [43]. The Westham Sarabi v2.1 ATSB does not currently have a target deployment density, nor is it known how spatial density of bait stations might interact with other environmental factors such as human settlement patterns, housing construction, the availability and location of vector larval sites or preferred natural sugar sources. Further research is needed to understand the importance of spatial bait station density and other factor to maximize intervention effectiveness in various malaria transmission settings.

## Conclusion

The more modest than expected impact of ATSB deployment on entomological outcomes observed in this trial was consistent with the epidemiological outcomes (Ashton et al*.*, in review). Non-significant trends suggesting moderate potential impact were evident in measures of vector density and biting, but not in the primary outcome of parity. Results observed in the Zambia ATSB trial may have been influenced by context-specific factors including the spatial density of bait stations driven by dispersed settlement patterns, environmental sources of natural sugar, as well as the bionomics of local populations of *An. funestus*, the dominant malaria vector in this setting. Additional cRCTs of the Westham Sarabi ATSB stations are currently underway in trial sites in Mali and Kenya. These trial settings differ in important ways from the Zambia trial site, including differences in population settlement patterns, dominant malaria vector species bionomics, levels of natural sugar availability, and climate. Results from these trials will provide additional evidence around the potential impact of ATSB to reduce malaria burden and important factors that may influence ATSB efficacy. Additional research will be needed to understand how to maximize the intervention impact in various transmission settings.

### Supplementary Information

Supplementary Material 1.

## Data Availability

De-identified data are available upon reasonable request. Following publication of forthcoming secondary analyses of trial data, the de-identified trial datasets will be available on a public repository.
