# Supplementary material for: Entomological effects of attractive targeted sugar bait station deployment in Western Zambia: vector surveillance findings from a two-arm cluster randomized phase III trial
Source: Malar J. 2024 Jul 18;23:214. doi: 10.1186/s12936-024-05045-3 (PMC11264679; doi:10.1186/s12936-024-05045-3)
Supplement: Supplementary file 1 — Supplementary Material 1. [file 12936_2024_5045_MOESM1_ESM.pptx]

## Slide 1
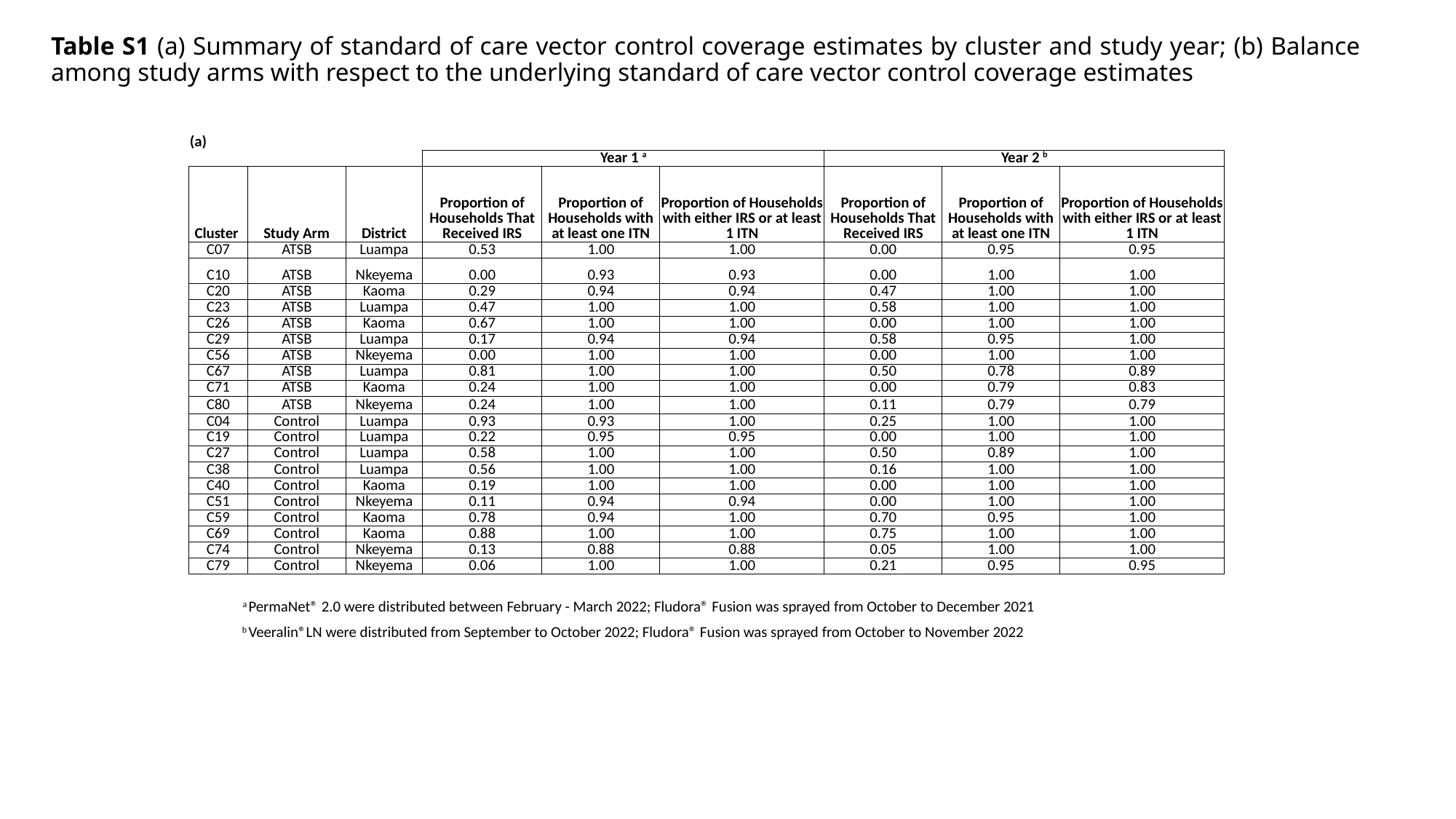

Table S1 (a) Summary of standard of care vector control coverage estimates by cluster and study year; (b) Balance among study arms with respect to the underlying standard of care vector control coverage estimates
| (a) | | | | | | | | |
| --- | --- | --- | --- | --- | --- | --- | --- | --- |
| | | | Year 1 a | | | Year 2 b | | |
| Cluster | Study Arm | District | Proportion of Households That Received IRS | Proportion of Households with at least one ITN | Proportion of Households with either IRS or at least 1 ITN | Proportion of Households That Received IRS | Proportion of Households with at least one ITN | Proportion of Households with either IRS or at least 1 ITN |
| C07 | ATSB | Luampa | 0.53 | 1.00 | 1.00 | 0.00 | 0.95 | 0.95 |
| C10 | ATSB | Nkeyema | 0.00 | 0.93 | 0.93 | 0.00 | 1.00 | 1.00 |
| C20 | ATSB | Kaoma | 0.29 | 0.94 | 0.94 | 0.47 | 1.00 | 1.00 |
| C23 | ATSB | Luampa | 0.47 | 1.00 | 1.00 | 0.58 | 1.00 | 1.00 |
| C26 | ATSB | Kaoma | 0.67 | 1.00 | 1.00 | 0.00 | 1.00 | 1.00 |
| C29 | ATSB | Luampa | 0.17 | 0.94 | 0.94 | 0.58 | 0.95 | 1.00 |
| C56 | ATSB | Nkeyema | 0.00 | 1.00 | 1.00 | 0.00 | 1.00 | 1.00 |
| C67 | ATSB | Luampa | 0.81 | 1.00 | 1.00 | 0.50 | 0.78 | 0.89 |
| C71 | ATSB | Kaoma | 0.24 | 1.00 | 1.00 | 0.00 | 0.79 | 0.83 |
| C80 | ATSB | Nkeyema | 0.24 | 1.00 | 1.00 | 0.11 | 0.79 | 0.79 |
| C04 | Control | Luampa | 0.93 | 0.93 | 1.00 | 0.25 | 1.00 | 1.00 |
| C19 | Control | Luampa | 0.22 | 0.95 | 0.95 | 0.00 | 1.00 | 1.00 |
| C27 | Control | Luampa | 0.58 | 1.00 | 1.00 | 0.50 | 0.89 | 1.00 |
| C38 | Control | Luampa | 0.56 | 1.00 | 1.00 | 0.16 | 1.00 | 1.00 |
| C40 | Control | Kaoma | 0.19 | 1.00 | 1.00 | 0.00 | 1.00 | 1.00 |
| C51 | Control | Nkeyema | 0.11 | 0.94 | 0.94 | 0.00 | 1.00 | 1.00 |
| C59 | Control | Kaoma | 0.78 | 0.94 | 1.00 | 0.70 | 0.95 | 1.00 |
| C69 | Control | Kaoma | 0.88 | 1.00 | 1.00 | 0.75 | 1.00 | 1.00 |
| C74 | Control | Nkeyema | 0.13 | 0.88 | 0.88 | 0.05 | 1.00 | 1.00 |
| C79 | Control | Nkeyema | 0.06 | 1.00 | 1.00 | 0.21 | 0.95 | 0.95 |
| | | | | | | | | |
| a | PermaNet® 2.0 were distributed between February - March 2022; Fludora® Fusion was sprayed from October to December 2021 | | | | | | | |
| b | Veeralin®LN were distributed from September to October 2022; Fludora® Fusion was sprayed from October to November 2022 | | | | | | | |

## Slide 2
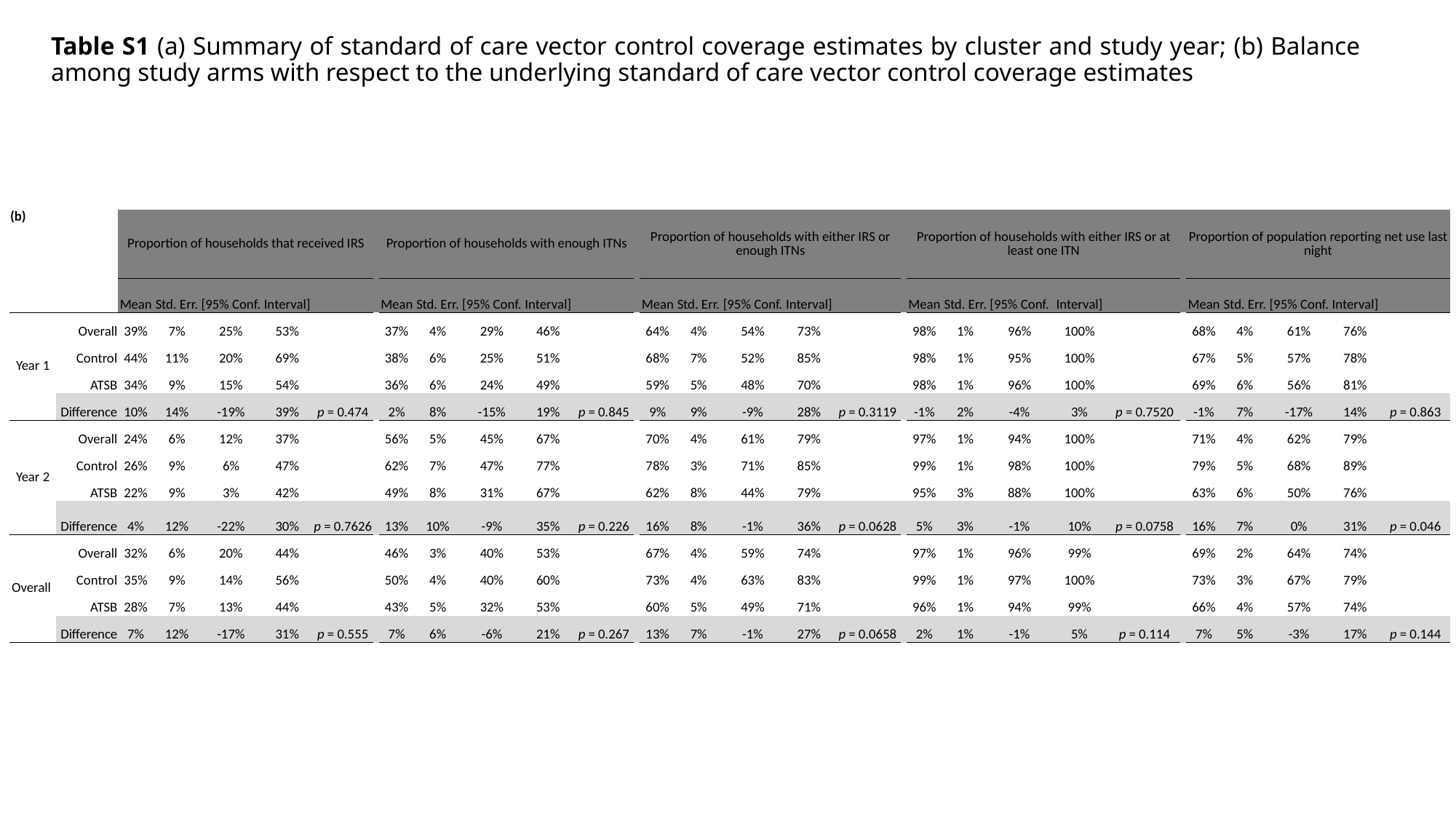

Table S1 (a) Summary of standard of care vector control coverage estimates by cluster and study year; (b) Balance among study arms with respect to the underlying standard of care vector control coverage estimates
| (b) | | Proportion of households that received IRS | | | | | | Proportion of households with enough ITNs | | | | | | Proportion of households with either IRS or enough ITNs | | | | | | Proportion of households with either IRS or at least one ITN | | | | | | Proportion of population reporting net use last night | | | | |
| --- | --- | --- | --- | --- | --- | --- | --- | --- | --- | --- | --- | --- | --- | --- | --- | --- | --- | --- | --- | --- | --- | --- | --- | --- | --- | --- | --- | --- | --- | --- |
| | | Mean | Std. Err. | [95% Conf. | Interval] | | | Mean | Std. Err. | [95% Conf. | Interval] | | | Mean | Std. Err. | [95% Conf. | Interval] | | | Mean | Std. Err. | [95% Conf. | Interval] | | | Mean | Std. Err. | [95% Conf. | Interval] | |
| Year 1 | Overall | 39% | 7% | 25% | 53% | | | 37% | 4% | 29% | 46% | | | 64% | 4% | 54% | 73% | | | 98% | 1% | 96% | 100% | | | 68% | 4% | 61% | 76% | |
| | Control | 44% | 11% | 20% | 69% | | | 38% | 6% | 25% | 51% | | | 68% | 7% | 52% | 85% | | | 98% | 1% | 95% | 100% | | | 67% | 5% | 57% | 78% | |
| | ATSB | 34% | 9% | 15% | 54% | | | 36% | 6% | 24% | 49% | | | 59% | 5% | 48% | 70% | | | 98% | 1% | 96% | 100% | | | 69% | 6% | 56% | 81% | |
| | Difference | 10% | 14% | -19% | 39% | p = 0.474 | | 2% | 8% | -15% | 19% | p = 0.845 | | 9% | 9% | -9% | 28% | p = 0.3119 | | -1% | 2% | -4% | 3% | p = 0.7520 | | -1% | 7% | -17% | 14% | p = 0.863 |
| Year 2 | Overall | 24% | 6% | 12% | 37% | | | 56% | 5% | 45% | 67% | | | 70% | 4% | 61% | 79% | | | 97% | 1% | 94% | 100% | | | 71% | 4% | 62% | 79% | |
| | Control | 26% | 9% | 6% | 47% | | | 62% | 7% | 47% | 77% | | | 78% | 3% | 71% | 85% | | | 99% | 1% | 98% | 100% | | | 79% | 5% | 68% | 89% | |
| | ATSB | 22% | 9% | 3% | 42% | | | 49% | 8% | 31% | 67% | | | 62% | 8% | 44% | 79% | | | 95% | 3% | 88% | 100% | | | 63% | 6% | 50% | 76% | |
| | Difference | 4% | 12% | -22% | 30% | p = 0.7626 | | 13% | 10% | -9% | 35% | p = 0.226 | | 16% | 8% | -1% | 36% | p = 0.0628 | | 5% | 3% | -1% | 10% | p = 0.0758 | | 16% | 7% | 0% | 31% | p = 0.046 |
| Overall | Overall | 32% | 6% | 20% | 44% | | | 46% | 3% | 40% | 53% | | | 67% | 4% | 59% | 74% | | | 97% | 1% | 96% | 99% | | | 69% | 2% | 64% | 74% | |
| | Control | 35% | 9% | 14% | 56% | | | 50% | 4% | 40% | 60% | | | 73% | 4% | 63% | 83% | | | 99% | 1% | 97% | 100% | | | 73% | 3% | 67% | 79% | |
| | ATSB | 28% | 7% | 13% | 44% | | | 43% | 5% | 32% | 53% | | | 60% | 5% | 49% | 71% | | | 96% | 1% | 94% | 99% | | | 66% | 4% | 57% | 74% | |
| | Difference | 7% | 12% | -17% | 31% | p = 0.555 | | 7% | 6% | -6% | 21% | p = 0.267 | | 13% | 7% | -1% | 27% | p = 0.0658 | | 2% | 1% | -1% | 5% | p = 0.114 | | 7% | 5% | -3% | 17% | p = 0.144 |

## Slide 3
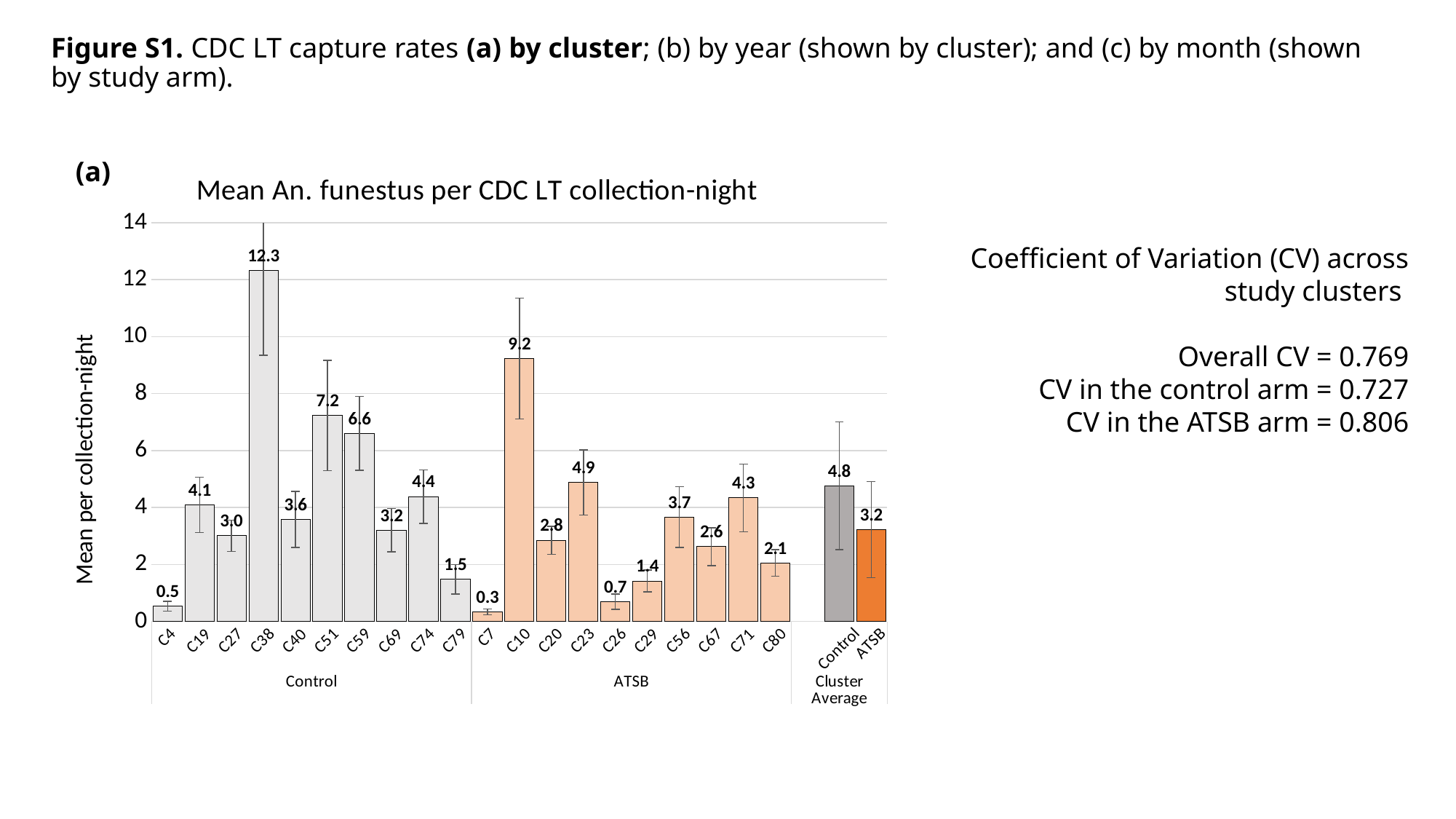

Figure S1. CDC LT capture rates (a) by cluster; (b) by year (shown by cluster); and (c) by month (shown by study arm).
### Chart: Mean An. funestus per CDC LT collection-night
| Category | |
|---|---|
| C4 | 0.5294118 |
| C19 | 4.092715 |
| C27 | 3.006757 |
| C38 | 12.31481 |
| C40 | 3.582677 |
| C51 | 7.231707 |
| C59 | 6.60119 |
| C69 | 3.208054 |
| C74 | 4.384146 |
| C79 | 1.47973 |
| C7 | 0.3355263 |
| C10 | 9.225806 |
| C20 | 2.846667 |
| C23 | 4.882716 |
| C26 | 0.6956522 |
| C29 | 1.42029 |
| C56 | 3.658537 |
| C67 | 2.624204 |
| C71 | 4.335878 |
| C80 | 2.052288 |
| | None |
| Control | 4.76206 |
| ATSB | 3.217991 |(a)
Coefficient of Variation (CV) across study clusters
Overall CV = 0.769
CV in the control arm = 0.727
CV in the ATSB arm = 0.806

## Slide 4
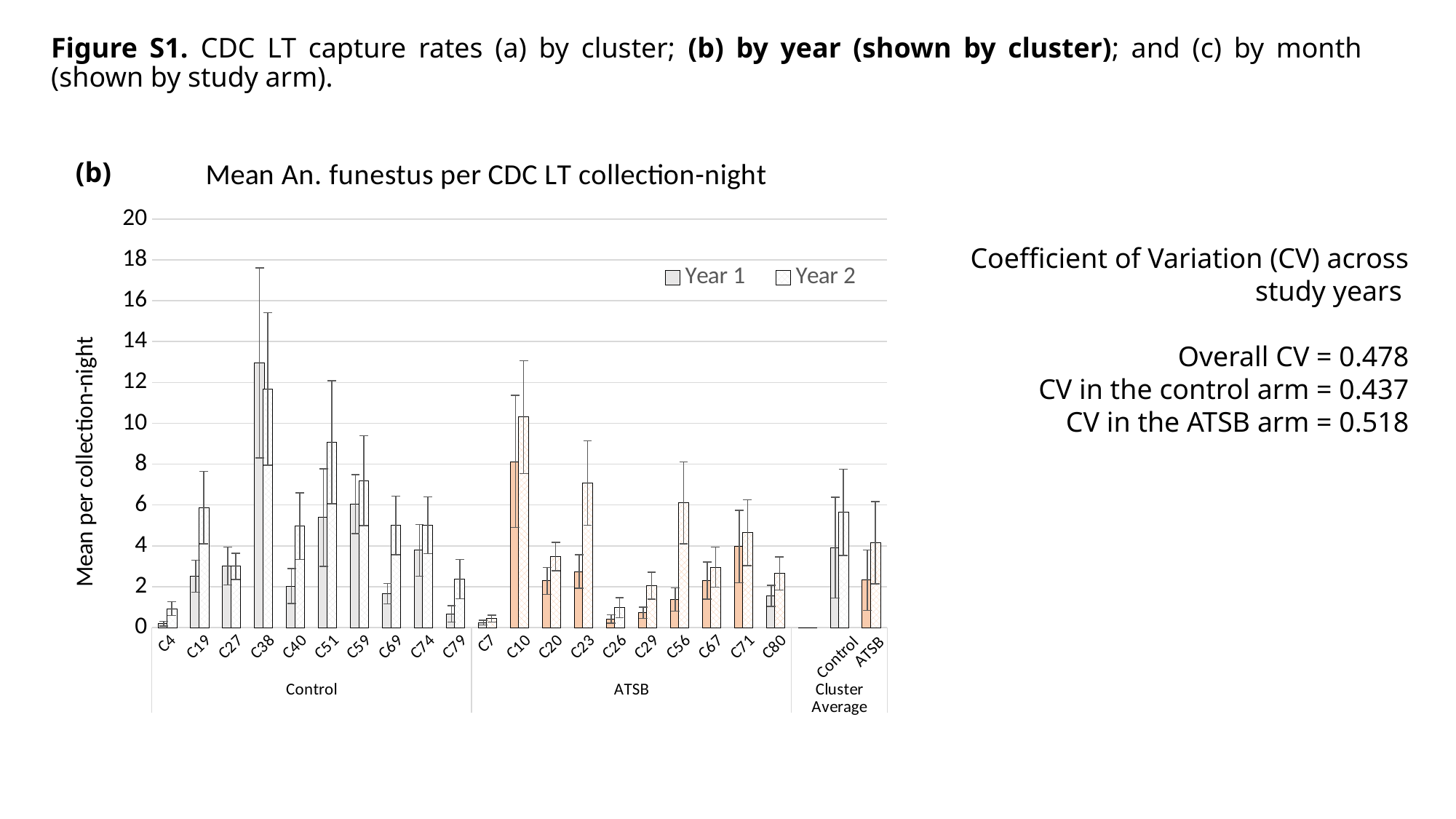

Figure S1. CDC LT capture rates (a) by cluster; (b) by year (shown by cluster); and (c) by month (shown by study arm).
### Chart: Mean An. funestus per CDC LT collection-night
| Category | Year 1 | Year 2 |
|---|---|---|
| C4 | 0.1927711 | 0.9285714 |
| C19 | 2.5125 | 5.873239 |
| C27 | 3.014925 | 3.0 |
| C38 | 12.95062 | 11.67901 |
| C40 | 2.033333 | 4.970149 |
| C51 | 5.390244 | 9.073171 |
| C59 | 6.045977 | 7.197531 |
| C69 | 1.6625 | 5.0 |
| C74 | 3.785714 | 5.0125 |
| C79 | 0.6794872 | 2.371429 |
| C7 | 0.2439024 | 0.4428571 |
| C10 | 8.12987 | 10.30769 |
| C20 | 2.2875 | 3.485714 |
| C23 | 2.743902 | 7.075 |
| C26 | 0.4197531 | 0.975 |
| C29 | 0.7272727 | 2.055556 |
| C56 | 1.376471 | 6.113924 |
| C67 | 2.308642 | 2.960526 |
| C71 | 3.966667 | 4.647887 |
| C80 | 1.559524 | 2.652174 |
| | 0.0 | 0.0 |
| Control | 3.915601 | 5.642287 |
| ATSB | 2.325193 | 4.150336 |(b)
Coefficient of Variation (CV) across study years
Overall CV = 0.478
CV in the control arm = 0.437
CV in the ATSB arm = 0.518

## Slide 5
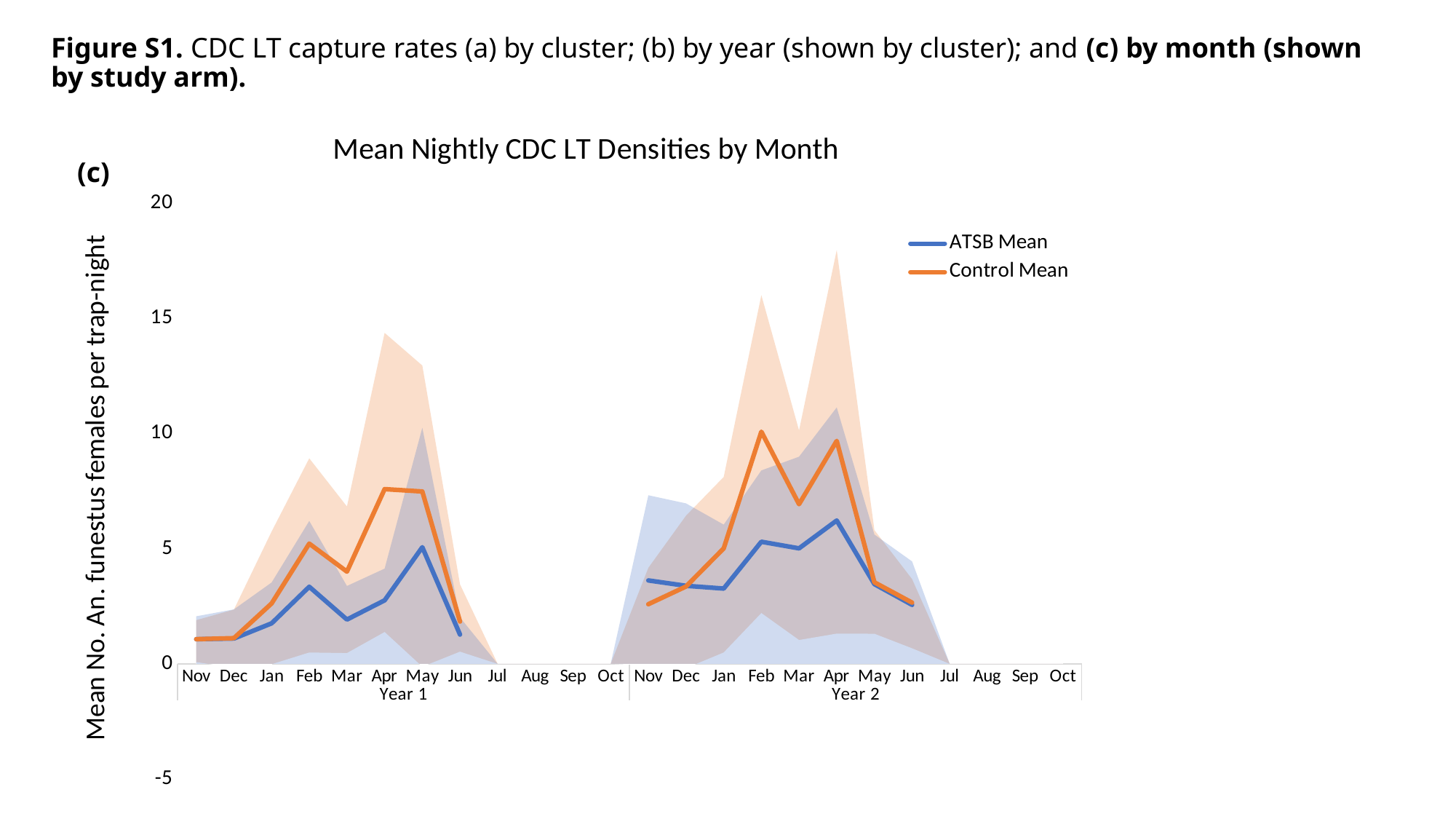

Figure S1. CDC LT capture rates (a) by cluster; (b) by year (shown by cluster); and (c) by month (shown by study arm).
### Chart: Mean Nightly CDC LT Densities by Month
| Category | ATSB | ATSB | Control | Control | ATSB | Control |
|---|---|---|---|---|---|---|
| Nov | 2.069819 | 0.07911749999999995 | 1.901418 | 0.24911989999999995 | 1.074468 | 1.075269 |
| Dec | 2.36993 | -0.16062789999999993 | 2.359754 | -0.1179958000000001 | 1.104651 | 1.120879 |
| Jan | 3.537081 | -0.012328200000000011 | 5.749934 | 0.0 | 1.762376 | 2.627451 |
| Feb | 6.209738 | 0.4961443000000001 | 8.930432 | 1.5185469999999999 | 3.352941 | 5.22449 |
| Mar | 3.385357 | 0.47031350000000005 | 6.838645 | 1.1817640000000003 | 1.927835 | 4.010204 |
| Apr | 4.139541 | 1.385212 | 14.38099 | 0.8071336999999996 | 2.762376 | 7.594059 |
| May | 10.26185 | -0.12043409999999977 | 12.96375 | 2.015842 | 5.070707 | 7.489796 |
| Jun | 2.018532 | 0.5324888 | 3.45664 | 0.22652859999999997 | 1.27551 | 1.841584 |
| Jul | None | None | None | None | None | None |
| Aug | None | None | None | None | None | None |
| Sep | None | None | None | None | None | None |
| Oct | None | None | None | None | None | None |
| Nov | 7.327249 | -0.07234719999999983 | 4.171398 | 1.015043 | 3.627451 | 2.59322 |
| Dec | 6.972529 | -0.18465070000000017 | 6.439171 | 0.28631930000000017 | 3.393939 | 3.362745 |
| Jan | 6.051161 | 0.4962073 | 8.122123 | 1.919114 | 3.273684 | 5.020619 |
| Feb | 8.40511 | 2.208751 | 16.02121 | 4.160612 | 5.306931 | 10.09091 |
| Mar | 9.002611 | 1.0377929999999997 | 10.14498 | 3.73257 | 5.020202 | 6.938776 |
| Apr | 11.1427 | 1.317298 | 17.97798 | 1.4020229999999998 | 6.23 | 9.69 |
| May | 5.619436 | 1.311258 | 5.81115 | 1.2797589999999999 | 3.465347 | 3.545455 |
| Jun | 4.452421 | 0.6788922 | 3.683366 | 1.643164 | 2.565657 | 2.663265 |
| Jul | None | None | None | None | None | None |
| Aug | None | None | None | None | None | None |
| Sep | None | None | None | None | None | None |
| Oct | None | None | None | None | None | None |(c)

## Slide 6
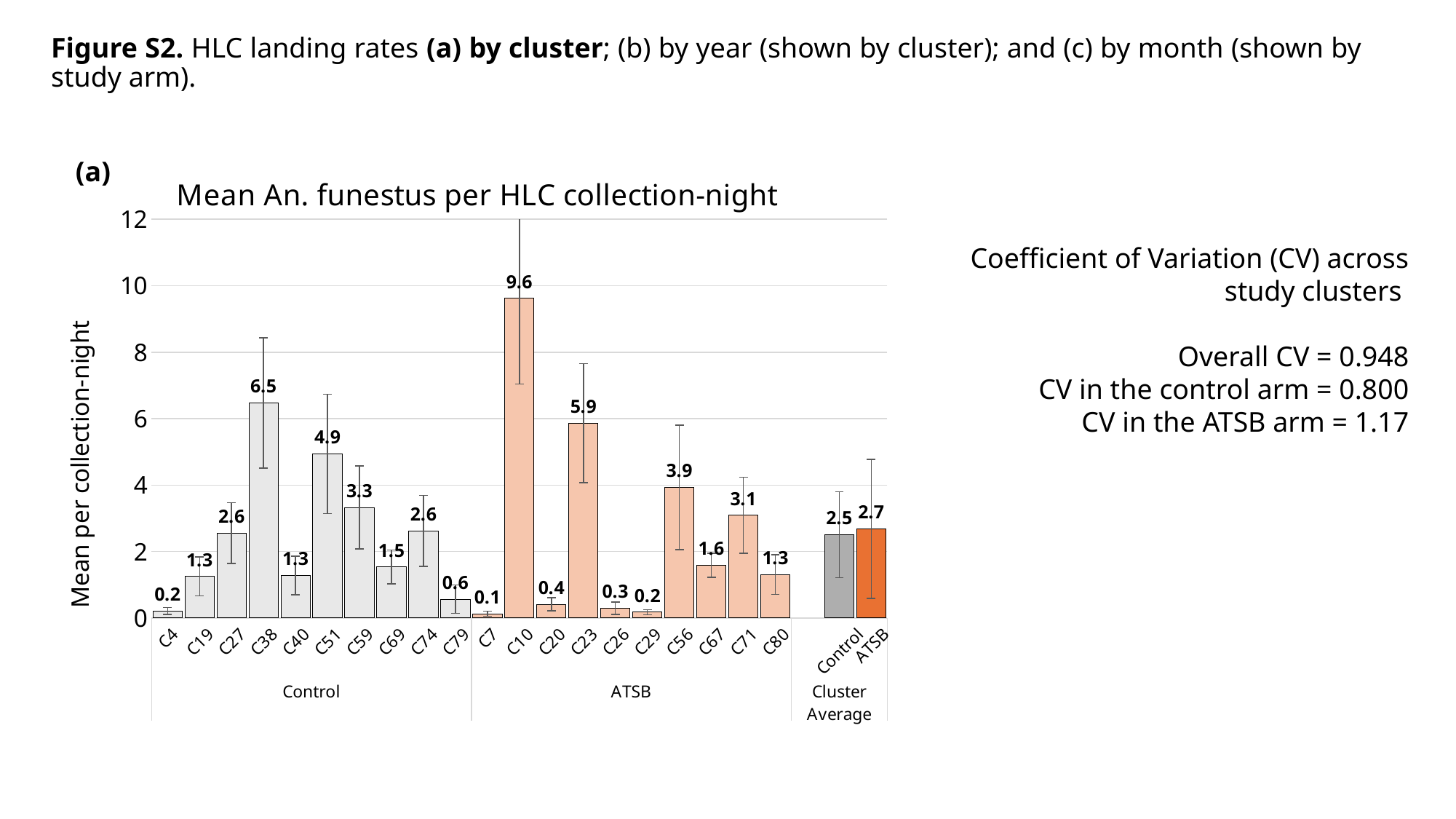

Figure S2. HLC landing rates (a) by cluster; (b) by year (shown by cluster); and (c) by month (shown by study arm).
### Chart: Mean An. funestus per HLC collection-night
| Category | |
|---|---|
| C4 | 0.2088608 |
| C19 | 1.253086 |
| C27 | 2.55625 |
| C38 | 6.473054 |
| C40 | 1.282258 |
| C51 | 4.935252 |
| C59 | 3.327684 |
| C69 | 1.5375 |
| C74 | 2.621951 |
| C79 | 0.5677419 |
| C7 | 0.1282051 |
| C10 | 9.619048 |
| C20 | 0.4150943 |
| C23 | 5.858896 |
| C26 | 0.2965116 |
| C29 | 0.1748252 |
| C56 | 3.934211 |
| C67 | 1.588957 |
| C71 | 3.090226 |
| C80 | 1.312102 |
| | None |
| Control | 2.505747 |
| ATSB | 2.686462 |(a)
Coefficient of Variation (CV) across study clusters
Overall CV = 0.948
CV in the control arm = 0.800
CV in the ATSB arm = 1.17

## Slide 7
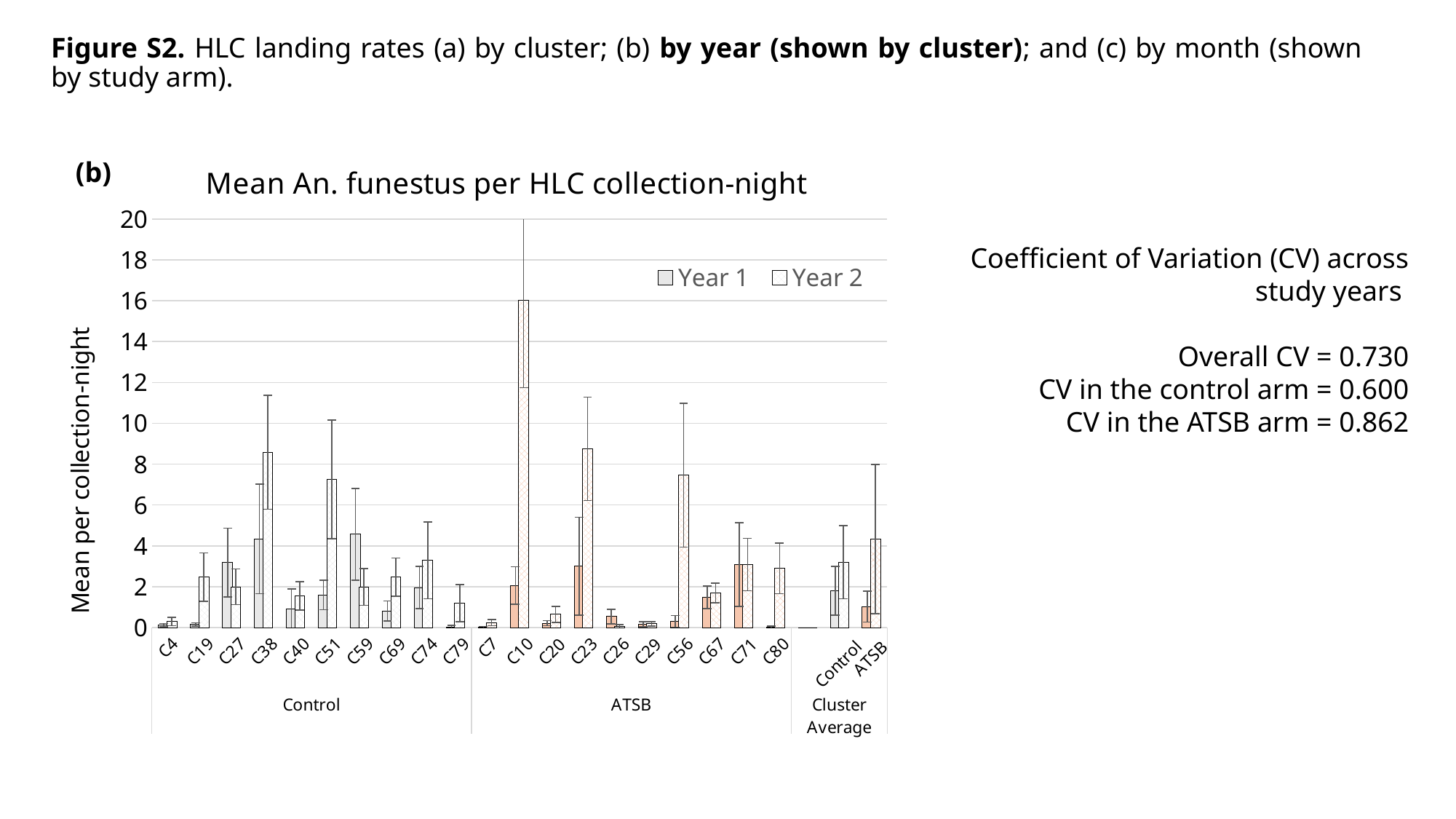

Figure S2. HLC landing rates (a) by cluster; (b) by year (shown by cluster); and (c) by month (shown by study arm).
### Chart: Mean An. funestus per HLC collection-night
| Category | Year 1 | Year 2 |
|---|---|---|
| C4 | 0.1204819 | 0.3066667 |
| C19 | 0.1529412 | 2.467532 |
| C27 | 3.186667 | 2.0 |
| C38 | 4.337349 | 8.583333 |
| C40 | 0.9038462 | 1.555556 |
| C51 | 1.596491 | 7.256098 |
| C59 | 4.565217 | 1.988235 |
| C69 | 0.8111111 | 2.471429 |
| C74 | 1.963855 | 3.296296 |
| C79 | 0.0357143 | 1.197183 |
| C7 | 0.0240964 | 0.2465753 |
| C10 | 2.064935 | 16.01099 |
| C20 | 0.2183908 | 0.6527778 |
| C23 | 3.0 | 8.753086 |
| C26 | 0.547619 | 0.0568182 |
| C29 | 0.1617647 | 0.1866667 |
| C56 | 0.3066667 | 7.467532 |
| C67 | 1.481481 | 1.695122 |
| C71 | 3.086207 | 3.093333 |
| C80 | 0.0344828 | 2.9 |
| | 0.0 | 0.0 |
| Control | 1.809949 | 3.203325 |
| ATSB | 1.033248 | 4.335459 |(b)
Coefficient of Variation (CV) across study years
Overall CV = 0.730
CV in the control arm = 0.600
CV in the ATSB arm = 0.862

## Slide 8
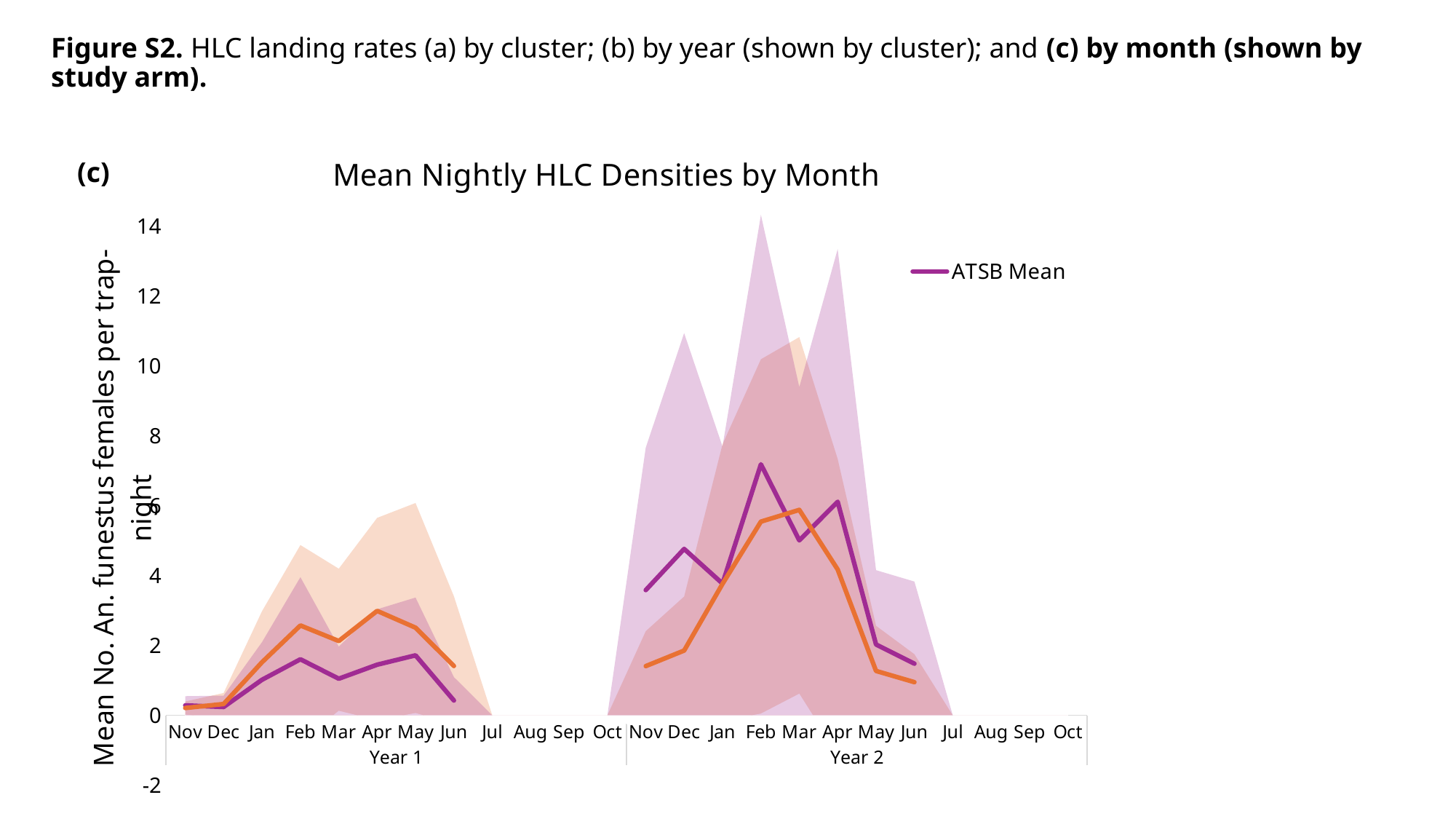

Figure S2. HLC landing rates (a) by cluster; (b) by year (shown by cluster); and (c) by month (shown by study arm).
### Chart: Mean Nightly HLC Densities by Month
| Category | ATSB | ATSB | Control | Control | ATSB | Control |
|---|---|---|---|---|---|---|
| Nov | 0.5492783 | 0.02215030000000001 | 0.3960708 | 0.02368229999999999 | 0.2857143 | 0.2098765 |
| Dec | 0.5648242 | -0.08755149999999998 | 0.6400447 | 0.009305999999999981 | 0.2386364 | 0.3246753 |
| Jan | 2.095042 | -0.05900630000000007 | 2.979433 | 0.0 | 1.018018 | 1.520408 |
| Feb | 3.956761 | -0.7462343 | 4.875448 | 0.27306680000000005 | 1.605263 | 2.574257 |
| Mar | 1.967491 | 0.12866270000000002 | 4.198356 | 0.05852499999999994 | 1.048077 | 2.12844 |
| Apr | 3.0368 | -0.13680019999999993 | 5.65426 | 0.32722169999999995 | 1.45 | 2.990741 |
| May | 3.372432 | 0.06446130000000005 | 6.0791 | -1.060232 | 1.718447 | 2.509434 |
| Jun | 1.095381 | -0.24689629999999996 | 3.409726 | -0.5828033000000001 | 0.4242424 | 1.413462 |
| Jul | None | None | None | None | None | None |
| Aug | None | None | None | None | None | None |
| Sep | None | None | None | None | None | None |
| Oct | None | None | None | None | None | None |
| Nov | 7.666948 | -0.4945341000000001 | 2.409768 | 0.4099041 | 3.586207 | 1.409836 |
| Dec | 10.94616 | -1.4167439999999996 | 3.402118 | 0.30661950000000004 | 4.764706 | 1.854369 |
| Jan | 7.697918 | -0.15336339999999993 | 7.761539 | -0.23212769999999994 | 3.772277 | 3.764706 |
| Feb | 14.32957 | 0.048809900000000184 | 10.19718 | 0.8971580999999995 | 7.189189 | 5.54717 |
| Mar | 9.400212 | 0.6184795999999997 | 10.83088 | 0.9338233000000002 | 5.009346 | 5.882353 |
| Apr | 13.35058 | -1.119809 | 7.344453 | 1.008489 | 6.115385 | 4.176471 |
| May | 4.156624 | -0.09837149999999983 | 2.564173 | -0.0221171 | 2.029126 | 1.271028 |
| Jun | 3.830077 | -0.8708931000000002 | 1.749855 | 0.14913540000000003 | 1.479592 | 0.9494949 |
| Jul | None | None | None | None | None | None |
| Aug | None | None | None | None | None | None |
| Sep | None | None | None | None | None | None |
| Oct | None | None | None | None | None | None |(c)

## Slide 9
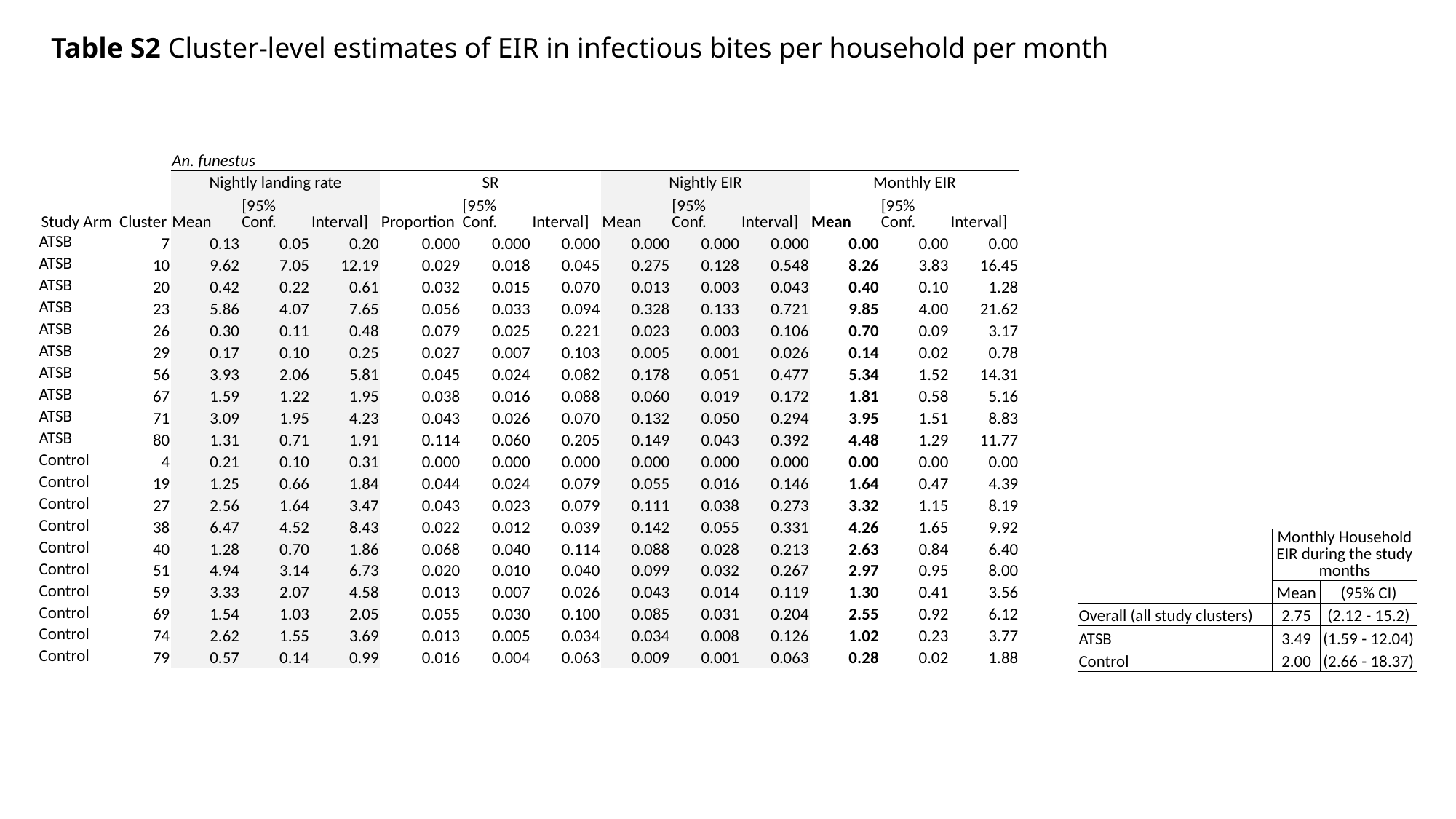

Table S2 Cluster-level estimates of EIR in infectious bites per household per month
| | | An. funestus | | | | | | | | | | | |
| --- | --- | --- | --- | --- | --- | --- | --- | --- | --- | --- | --- | --- | --- |
| | | Nightly landing rate | | | SR | | | Nightly EIR | | | Monthly EIR | | |
| Study Arm | Cluster | Mean | [95% Conf. | Interval] | Proportion | [95% Conf. | Interval] | Mean | [95% Conf. | Interval] | Mean | [95% Conf. | Interval] |
| ATSB | 7 | 0.13 | 0.05 | 0.20 | 0.000 | 0.000 | 0.000 | 0.000 | 0.000 | 0.000 | 0.00 | 0.00 | 0.00 |
| ATSB | 10 | 9.62 | 7.05 | 12.19 | 0.029 | 0.018 | 0.045 | 0.275 | 0.128 | 0.548 | 8.26 | 3.83 | 16.45 |
| ATSB | 20 | 0.42 | 0.22 | 0.61 | 0.032 | 0.015 | 0.070 | 0.013 | 0.003 | 0.043 | 0.40 | 0.10 | 1.28 |
| ATSB | 23 | 5.86 | 4.07 | 7.65 | 0.056 | 0.033 | 0.094 | 0.328 | 0.133 | 0.721 | 9.85 | 4.00 | 21.62 |
| ATSB | 26 | 0.30 | 0.11 | 0.48 | 0.079 | 0.025 | 0.221 | 0.023 | 0.003 | 0.106 | 0.70 | 0.09 | 3.17 |
| ATSB | 29 | 0.17 | 0.10 | 0.25 | 0.027 | 0.007 | 0.103 | 0.005 | 0.001 | 0.026 | 0.14 | 0.02 | 0.78 |
| ATSB | 56 | 3.93 | 2.06 | 5.81 | 0.045 | 0.024 | 0.082 | 0.178 | 0.051 | 0.477 | 5.34 | 1.52 | 14.31 |
| ATSB | 67 | 1.59 | 1.22 | 1.95 | 0.038 | 0.016 | 0.088 | 0.060 | 0.019 | 0.172 | 1.81 | 0.58 | 5.16 |
| ATSB | 71 | 3.09 | 1.95 | 4.23 | 0.043 | 0.026 | 0.070 | 0.132 | 0.050 | 0.294 | 3.95 | 1.51 | 8.83 |
| ATSB | 80 | 1.31 | 0.71 | 1.91 | 0.114 | 0.060 | 0.205 | 0.149 | 0.043 | 0.392 | 4.48 | 1.29 | 11.77 |
| Control | 4 | 0.21 | 0.10 | 0.31 | 0.000 | 0.000 | 0.000 | 0.000 | 0.000 | 0.000 | 0.00 | 0.00 | 0.00 |
| Control | 19 | 1.25 | 0.66 | 1.84 | 0.044 | 0.024 | 0.079 | 0.055 | 0.016 | 0.146 | 1.64 | 0.47 | 4.39 |
| Control | 27 | 2.56 | 1.64 | 3.47 | 0.043 | 0.023 | 0.079 | 0.111 | 0.038 | 0.273 | 3.32 | 1.15 | 8.19 |
| Control | 38 | 6.47 | 4.52 | 8.43 | 0.022 | 0.012 | 0.039 | 0.142 | 0.055 | 0.331 | 4.26 | 1.65 | 9.92 |
| Control | 40 | 1.28 | 0.70 | 1.86 | 0.068 | 0.040 | 0.114 | 0.088 | 0.028 | 0.213 | 2.63 | 0.84 | 6.40 |
| Control | 51 | 4.94 | 3.14 | 6.73 | 0.020 | 0.010 | 0.040 | 0.099 | 0.032 | 0.267 | 2.97 | 0.95 | 8.00 |
| Control | 59 | 3.33 | 2.07 | 4.58 | 0.013 | 0.007 | 0.026 | 0.043 | 0.014 | 0.119 | 1.30 | 0.41 | 3.56 |
| Control | 69 | 1.54 | 1.03 | 2.05 | 0.055 | 0.030 | 0.100 | 0.085 | 0.031 | 0.204 | 2.55 | 0.92 | 6.12 |
| Control | 74 | 2.62 | 1.55 | 3.69 | 0.013 | 0.005 | 0.034 | 0.034 | 0.008 | 0.126 | 1.02 | 0.23 | 3.77 |
| Control | 79 | 0.57 | 0.14 | 0.99 | 0.016 | 0.004 | 0.063 | 0.009 | 0.001 | 0.063 | 0.28 | 0.02 | 1.88 |
| | Monthly Household EIR during the study months | |
| --- | --- | --- |
| | Mean | (95% CI) |
| Overall (all study clusters) | 2.75 | (2.12 - 15.2) |
| ATSB | 3.49 | (1.59 - 12.04) |
| Control | 2.00 | (2.66 - 18.37) |
